# High Pressure Brillouin Spectroscopy and X-ray Diffraction of Cerium Dioxide

**DOI:** 10.3390/ma14133683

**Published:** 2021-07-01

**Authors:** Mungo Frost, John D. Lazarz, Abraham L. Levitan, Vitali B. Prakapenka, Peihao Sun, Sergey N. Tkachev, Hong Yang, Siegfried H. Glenzer, Arianna E. Gleason

**Affiliations:** 1 SLAC National Accelerator Laboratory, 2575 Sand Hill Road, Menlo Park, CA 94025, USA; alevitan@mit.edu (A.L.L.); phsun@stanford.edu (P.S.); glenzer@slac.stanford.edu (S.H.G.); ariannag@stanford.edu (A.E.G.); 2 Los Alamos National Laboratory, P.O. BOX 1663, Los Alamos, NM 87545, USA; lazarz.john@gmail.com; 3 Center for Advanced Radiation Sources, University of Chicago, Chicago, IL 60637, USA; prakapenka@cars.uchicago.edu (V.B.P.); tkachev@cars.uchicago.edu (S.N.T.); 4 Department of Geological Sciences, Stanford University, Stanford, CA 94305, USA; hyang666@stanford.edu

**Keywords:** cerium dioxide, ceria, high pressure, diamond anvil cell, brillouin spectroscopy

## Abstract

Simultaneous high-pressure Brillouin spectroscopy and powder X-ray diffraction of cerium dioxide powders are presented at room temperature to a pressure of 45 GPa. Micro- and nanocrystalline powders are studied and the density, acoustic velocities and elastic moduli determined. In contrast to recent reports of anomalous compressibility and strength in nanocrystalline cerium dioxide, the acoustic velocities are found to be insensitive to grain size and enhanced strength is not observed in nanocrystalline CeO${}_{2}$. Discrepancies in the bulk moduli derived from Brillouin and powder X-ray diffraction studies suggest that the properties of CeO${}_{2}$ are sensitive to the hydrostaticity of its environment. Our Brillouin data give the shear modulus, $G_{0}$ = 63 (3) GPa, and adiabatic bulk modulus, $K_{S0}$ = 142 (9) GPa, which is considerably lower than the isothermal bulk modulus, $K_{T0}∼$ 230 GPa, determined by high-pressure X-ray diffraction experiments.

## 1. Introduction

Brillouin spectroscopy allows for the direct determination of acoustic velocities and elastic moduli of materials, and is ideally suited for measuring these at high-pressures in diamond anvil cells [1]. It is well complemented by X-ray diffraction which allows a direct measurement of the density of the compressed material. Both techniques may be applied to either single- or poly-crystalline samples. While direction dependent elastic moduli may be determined from a single crystal, polycrystalline samples are of interest in the study of bulk properties and material response arising from both crystalline cores and grain boundaries. Powder samples also avoid the difficulty of maintaining single crystals to high pressure.

Cerium dioxide, CeO${}_{2}$, has a number of uses including catalysis [2], sensors [3], and an emerging application as an oxygen ion conductor in solid oxide fuel cells [4]. It is also widely used as a non-hazardous analogue for the development of ceramic nuclear fuels, where its physical properties mimic those of oxide nuclear fuels [5,6,7]. This allows testing of designs without the hazards associated with radioactive compounds. Procedures for end of life disposal of spent fuel are vital for future nuclear energy. Most proposed methods to deal with spent nuclear fuel involve its long-term entombment in the Earth [8]. Understanding its polycrystalline high-pressure behavior is vital where deep storage systems may collapse or be subject to seismic activity.

At ambient conditions cerium dioxide adopts a cubic fluorite structure with space group $Fm\bar{3}m$. This persists to 31.5 GPa where it transforms to an α-PbCl${}_{2}$ type structure with the Pnam space group [9,10]. This transition is kinetically slow with the low-pressure phase co-existing substantially above the transition pressure. Hysteresis is also observed on decompression with the high-pressure α-PbCl${}_{2}$-type structure persisting considerably below the nominal 31.5 GPa transition pressure [9,11,12]. The bulk modulus of the low-pressure fluorite phase of cerium dioxide is the subject of considerable disagreement in the literature. A number of studies report the compression of powdered cerium dioxide in diamond anvil cells and find bulk moduli of 230 [9], 235 [13], and 220 GPa [14]. The studies reporting higher $K_{0}$ values did not utilize pressure transmitting media (PTM) and so will shows the effect of non-hydrostatic strain. Liu et al. [13] attempted to correct for this using a combination of line-shift and line-width analysis, and report non-hydrostaticity increases from 1 to 2 GPa between measured pressures of 2 and 25 GPa. Other studies have also investigated the extraction of equations of state by applying corrections to data collected from non-hydrostatically compressed samples [15,16]. Gerward et al. [14] compressed to 20 GPa with a 16:3:1 methanol:ethanol:water PTM which is hydrostatic below 10 GPa, though quite stiff above this [17], and find a lower bulk modulus of 220 GPa.

Recently, it has been reported that the compressibility of cerium dioxide depends on the grain size of the powder, with nanoparticles exhibiting different behavior from microcrystalline samples. Below 10 GPa the bulk modulus of nanocrystalline cerium dioxide measured by high pressure diffraction varies considerably with reported values ranging between 248 and 328 GPa [18,19,20]. These values are higher than the value for bulk cerium dioxide. A summary of literature values for the bulk modulus of cerium dioxide is presented in Table 1.

Above 15 GPa a dramatic decrease in the compressibility of cerium dioxide nanopowder is reported, attributed to a ‘core-shell’ model [18]. A plateau is reported in the compressibility curve of cerium dioxide between 15 and 25 GPa, the exact nature of which depends on the PTM. For silicone oil a negative bulk modulus is reported in this region, which violates the stability criteria for crystals [22]. It is also worth noting that above 15 GPa a silicone oil PTM is known to become substantially non-hydrostatic [17].

The uncertainty in the literature is compounded when previous acoustic measurements are considered. To date, this is the only Brillouin study performed on cerium dioxide at high pressure, but ambient pressure measurements have been reported [23]. From these the elastic constants and adiabatic bulk modulus can be calculated. The bulk modulus is found to be 204 GPa, anomalously lower than the values for the isothermal bulk modulus reported by high pressure powder X-ray diffraction. It should be noted that thermodynamically the adiabatic bulk modulus must be greater than or equal to the isothermal bulk modulus [24,25].

The elasticity of cerium dioxide has also been explored by density functional theory [14,26,27,28,29,30]. The bulk moduli thence obtained vary depending on the details of the simulation, and have been reported between 177 [14] and 236 GPa [26], with results based on the local density approximation (LDA) generally higher, in the region of 210 GPa, than those obtained via the generalized gradient approximation (GGA), yielding values around 180 GPa. In general, theoretical results lie closer to the bulk modulus measured via Brillouin spectroscopy [23] than those reported from high-pressure powder X-ray diffraction [9,13,14]. These results are summarized in Table 2.

Despite the large body of literature on the compressibility of cerium dioxide, few clear trends are present. The choice of pressure transmitting media, the crystallite size, nanoparticle shape and degree of crystallinity influence elasticity and measured compressibility [31,32]. In addition to X-ray diffraction cerium dioxide has also been studied via high-pressure Raman spectroscopy [11,12,33,34,35] and under shock conditions [36,37]. Here, we investigate micro- and nanocrystalline cerium dioxide using simultaneous powder X-ray diffraction and Brillouin spectroscopy to determine the high pressure elastic moduli. The density, sound velocities and elastic moduli are measured to 45 GPa without a pressure transmitting medium.

## 2. Materials and Methods

Cerium dioxide powders of nano- and micro-scale grain size were loaded without a PTM into diamond anvil cells equipped with wide angle Bohler—Almax anvils with 300 μm diameter culets. The nanopowder (Sigma-Adrich, St. Louis, MO, USA) had grain size <25 nm, while the micropowder (Sigma-Aldrich, 99.9% trace metals basis) had a typical grain size of 5 μm. Rhenium gaskets were indented to 50 μm thickness and sample holes drilled using a laser cutting apparatus [38]. Pressure was determined using ruby fluorescence [39] and/or the equation of state of a small flake of gold [40] included in the loading.

Brillouin spectroscopy and powder X-ray diffraction were performed at GSECARS 13-BM-D at the Advanced Photon Source. Both techniques probed the same location on the sample. Angle dispersive powder X-ray diffraction was performed using 0.3344 Å radiation focussed to a 6 × 12 mm spot. Diffraction patterns were collected on a PerkinElmer amorphous silicon flat panel X-ray detector. Patterns were integrated using the Dioptas software package [41] and LeBail fits performed using Jana [42].

Brillouin spectra were collected using a 532 nm frequency doubled Nd:YVO${}_{4}$ excitation laser with a six pass Fabry–Pérot interferometer [43]. Equal angle geometry (θ = 50 ${}^{\circ }$) was used to obviate the need to know the refractive index under pressure [43]. The intensity of the Brillouin modes observed was very low, requiring 2 to 3 h collection times to achieve clear peaks. Even with such collections the longitudinal mode could not always be observed above background.

Compression was performed without a PTM to optimize the Brillouin signal. Compression to 2 GPa results in greater transparency compared to pre-pressed samples embedded in a PTM. The reduced transparency when compressing with a PTM, presence of multiple reflections from PTM-CeO${}_{2}$ interfaces, and additional Brillouin features from the PTM, makes the use of a PTM impractical in this case.

## 3. Results

**Figure 1:**
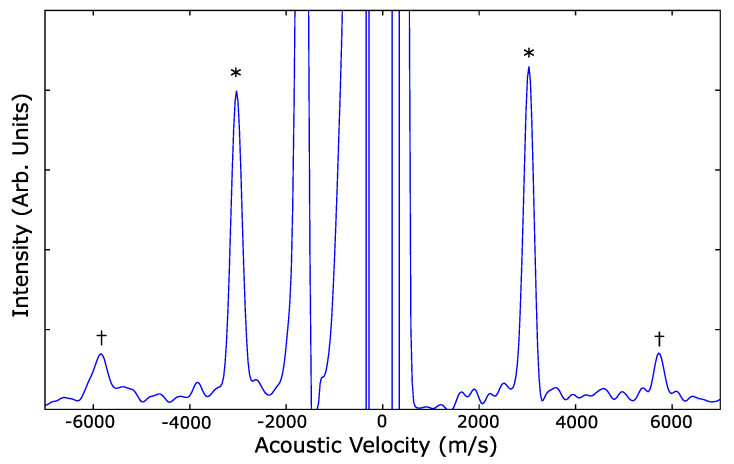
Brillouin spectrum of cerium dioxide nanopowder at 3.6 GPa. Transverse modes are marked with asterisks, longitudinal modes occur at higher shift and are marked with daggers.

**Figure 2:**
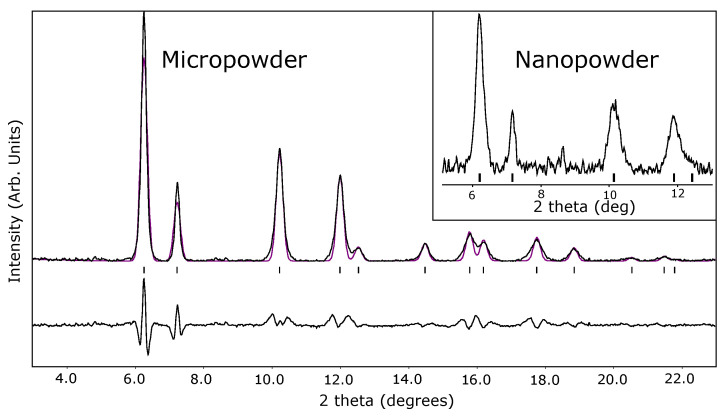
Powder X-ray diffraction pattern of cerium dioxide micropowder at 17.9 GPa collected using 0.3344 Å radiation. Integrated data in black with LeBail fit in red and residual below. Tics show angles of allowed reflections of low-pressure $Fm\bar{3}m$ phase. **Inset:** Integrated pattern from nanopowder at 12.0 GPa, the peaks are broadened due to size effects. Additional diffraction patterns are shown in the supplemental materials.

The Brillouin spectrum of cerium dioxide at 3.6 GPa (Figure 1) shows a weak longitudinal mode compared to the transverse mode. Figure 2 shows a representative powder X-ray diffraction pattern of the cerium dioxide at 17.9 GPa which was used to calculate the in situ density.

**Figure 3:**
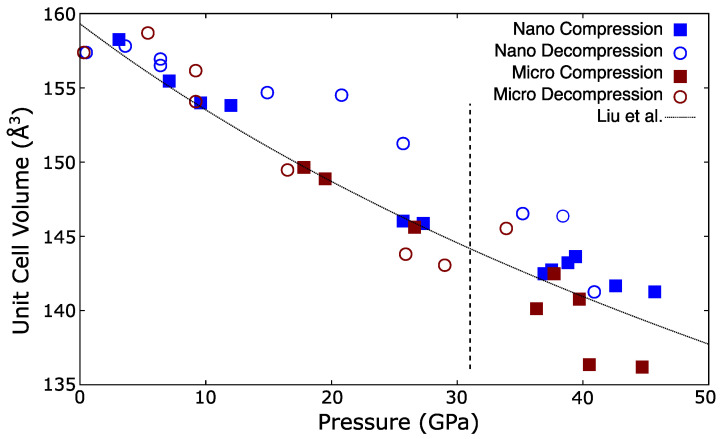
Unit cell volume vs. pressure for $Fm\bar{3}m$ cerium dioxide micro- and nanopowders on compression and decompression. Vertical dashed line indicates nominal transition pressure from the $Fm\bar{3}m$ phase to Pnam, note that the $Fm\bar{3}m$ phase persists above this. Uncertainties from LeBail fits are typically around 0.1 Å${}^{3}$, and smaller than plotting points.

The experimentally determined unit cell volumes of the low-pressure $Fm\bar{3}m$ phase of cerium dioxide micro- and nanopowders are shown in Figure 3 along with the equation of state fitted by Liu et al. [13] without line width corrections. This non-hydrostatic equation of state agrees well with the values observed here. The scatter in the unit cell volumes vs pressure in Figure 3 arises from differing non-hydrostatic strain which varied between samples, with pressure, and on compression and decompression.

On decompression the unit cell volume of the nanopowder was anomalously large, particularly between 15 and 25 GPa. This could arise from mechanisms involving differing behavior between the core and shell of the nanoparticles as has been proposed in previous compression studies of cerium dioxide nanoparticles [18,19]. However, on compression the unit cell volume of the nanopowder agreed with that of the micropowder, which would not be expected were it actually stiffer. Therefore non-hydrostaticity could not be ruled out as the cause of this discrepancy.

**Figure 4:**
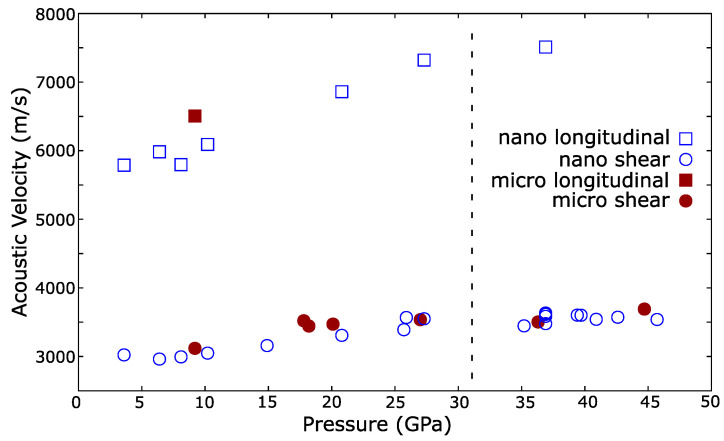
The evolution of the sound velocities of cerium dioxide micro- and nanopowders with pressure. Open symbols are nanopowders, closed symbols are micropowders. Squares are longitudinal mode velocities, circles are transverse. Acoustic velocity is not significantly affected by particle size. Vertical dashed line indicates the nominal transition pressure from the $Fm\bar{3}m$ phase to Pnam. Uncertainties are of similar size to plotting points: 40 ms${}^{-1}$ for shear and 60 ms${}^{-1}$ for longitudinal sound velocities.

A plot of the sound velocities of the micro- and nanopowders as a function of pressure is shown in Figure 4. Both sets of data lay on similar trends suggesting that the sound velocity was insensitive to grain size.

**Figure 5:**
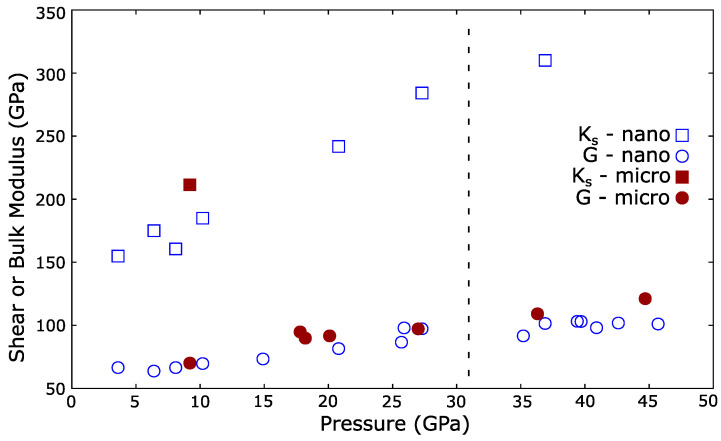
Adiabatic bulk moduli ($K_{s}$, squares) and shear moduli (*G*, circles) of cerium dioxide nano- and micropowders (open and closed symbols respectively) as a function of pressure. Vertical dashed line indicates the nominal transition pressure from the $Fm\bar{3}m$ phase to Pnam.

Using the longitudinal sound velocity, $V_{p}$, and transverse sound velocity, $V_{s}$, measured by Brillouin spectroscopy and the mass density, ρ, measured in situ by powder X-ray diffraction, it is possible to calculate the elastic moduli of the material via:

(1) $$K_{s}=\rho \left(V_{p}^{2}-\frac{4V_{s}^{2}}{3}\right)$$

(2) $$G=\rho V_{s}^{2}$$

where $K_{s}$ and *G* are the adiabatic bulk modulus and shear modulus respectively. The pressure dependences of $K_{s}$ and *G* for micro- and nanopowders are shown in Figure 5. The shear modulus was insensitive to particle size and increases with pressure. Increasing *G* with pressure is normal and observed in other materials [1,44]. As noted, the longitudinal mode was extremely weak and was only detected at one pressure from the micropowder. However, this lay on the established trend observed in the nanopowder suggesting that the adiabatic bulk modulus was similarly insensitive to grain size, in agreement with the densities determined via X-ray diffraction, see Figure 3.

Above 31.5 GPa cerium dioxide is known to undergo a phase transition from the low-pressure cubic fluorite structure to a high-pressure *Pnam* α-PbCl${}_{2}$-type structure [9,10]. The results here on the micropowder agree with the literature. While some transformation to the high pressure Pnam phase occurred by 36 GPa, some of the low pressure fluorite structure persisted to the highest pressure reached. A similar effect was observed on decompression with traces of the Pnam phase observable to 5 GPa. Other studies also report a significant pressure range of coexistence [9,10]. Only a small quantity of the Pnam phase formed in the nanopowder at 38 GPa on decompression with traces persisting to 6 GPa. The density of the high pressure phase was about 8% greater than the low pressure one, within the range of values reported previously [9,10].

## 4. Discussion

Various recent studies have observed anomalous compressibility in cerium dioxide nanoparticles [18,19,45]. The effect is strongly dependent on the PTM used, nanoparticle size and the influence of the PTM on the nano-grain boundaries. Cerium dioxide nanoparticles are reported to show an anomalously low compressibility at low pressure, with the bulk modulus reported to exceed the bulk value by 25 to 40% [18,19]. Our observation that the sound velocities are similar between micro- and nanopowders, Figure 4 and Figure 5, is incompatible with this and suggests that the strengths of the materials are similar under pressure. The reported anomalous behavior is dependent on the PTM used. Coupled with our results, this suggests that the observations may be due to changes in the non-hydrostatic strain on the sample as PTMs harden at high pressure [17], or from more complex interactions between the nanoparticles and PTM, and are not representative of cerium dioxide alone under hydrostatic strain.

The adiabatic bulk moduli measured under pressure, see Figure 5, are rather lower than the isothermal bulk moduli reported from powder X-ray diffraction which fit the zero pressure bulk modulus between 220 and 235 GPa [13,14]. However, the elastic moduli reported from Brillouin scattering at ambient pressure more closely agree with those observed here [23]. The literature zero-pressure shear modulus is reported to be 60 GPa, in agreement with $G_{0}$ = 63 (3) GPa extrapolated to zero-pressure from our data. The reported zero-pressure adiabatic bulk modulus is 204 GPa. This value is higher than that observed here under non-hydrostatic conditions, which extrapolates to $K_{S0}$ = 142 (9) GPa. Both values are considerably lower than the isothermal bulk modulus fitted from high pressure powder X-ray diffraction experiments. It should be noted that the data here are from cerium dioxide compressed without a pressure transmitting medium so there will be a few GPa of non-hydrostaticity [13]. Lowering of the acoustic velocities by non-hydrostatic stress has been observed in magnesium oxide [31], and may explain the low adiabatic bulk modulus obtained in our Brillouin measurements.

This highlights a substantial discrepancy in the literature as the adiabatic bulk modulus is strictly greater than the isothermal bulk modulus. [25]. The origin of the disagreement between the isothermal zero-pressure bulk modulus of cerium dioxide measured using powder X-ray diffraction and the adiabatic bulk modulus measured using Brillouin scattering is like attributable to non-hydrostaticity at high-pressure. The X-ray powder diffraction studies which report the highest bulk moduli, around 235 GPa, [9,13] compressed without a pressure transmitting medium which results in large non-hydrostaticity. This causes difficulties in fitting equations of state to obtain a zero-pressure value for $K_{T}$. A study utilizing a PTM reports a lower bulk modulus of 220 GPa [14] but their data run to 20 GPa which is significantly in excess of the hydrostatic limit of their PTM [17]. Along with the insensitivity of the elastic moduli to grain size measured using Brillouin spectroscopy, this suggests caution must be taken when considering compressibility of nanoparticles at pressures where there are changes in the hardness of the pressure transmitting media used.

## 5. Conclusions

In conclusion, we have compared the acoustic velocities of micro- and nano-crystalline cerium dioxide under pressure and do not observe any significant difference between them despite reports of anomalous compressibility in cerium dioxide nanoparticles. The adiabatic and shear moduli derived from the acoustic velocities are reported as a function of pressure. The shear modulus agrees well with the value measured at zero pressure while the adiabatic bulk modulus is lower than the isothermal bulk modulus reported from high-pressure powder X-ray diffraction experiment. The disparity between elastic moduli measured using Brillouin scattering and high-pressure powder X-ray diffraction arises from non-hydrostaticity suggesting that extreme care must be taken in selection of pressure transmitting media when considering the compressibility of cerium dioxide nanoparticles.

## Figures and Tables

**Table 1:** Bulk moduli of $Fm\bar{3}m$ cerium dioxide measured using X-ray diffraction in diamond anvil cells. All studies use ruby fluorescence to determine pressure, except Wang 2004 which uses the equation of state of Pt, and Wang 2014 which uses both ruby and the equation of state of Au. ‘BM’ refers to the Birch-Murnaghan equation of state.

Ref.	PTM	Grain Size	$K_{0}$ (GPa)	$K_{0}^{\prime }$	P${}_{\max}$ (GPa)	EoS Form
Duclos [9]	None	Not Specified	230 (10)	4 (fixed)	38	Birch
Gerward [14]	16:3:1 MeOH:EtOH:H${}_{2}$O	‘Finely Ground’	220 (9)	4.4 (4)	20	BM
Liu 2012 [13]	None (with corrections)	5 μm	235 (18)	3.67	27	Vinet
Liu 2012 [13]	None (no corrections)	5 μm	248 (52)	4.56	27	Vinet
Liu 2011 [21]	4:1 MeOH:EtOH	150 nm	260 (10)	4 (fixed)	55	Vinet
Wang 2014 [18]	Various	12 nm	287 (5)	4 (fixed)	16	BM
Wang 2004 [19]	None	10 nm	328 (12)	4 (fixed)	20	BM
Ge [20]	16:3:1 MeOH:EtOH:H${}_{2}$O	4.7 nm	230		28	

**Table 2:** Bulk moduli of $Fm\bar{3}m$ cerium dioxide as determined by theory.

Ref.	Method	$K_{0}$ (GPa)	$K_{0}^{\prime }$	EoS Form
Gerward [14]	LDA	176.9		BM
Mehrotra [26]	LDA	236		Birch
Skorodumova [27]	LDA	214.7		
Skorodumova [27]	GGA	187.7		
Gurel [30]	LDA	210.1	4.4	Vinet
Kanchana [28]	LDA	218	4.2	Birch
Kanchana [28]	GGA	184	4.2	Birch
Fabris [29]	LDA	210.7		
Fabris [29]	GGA	178		

## Data Availability

Data are available on reasonable request.

## Supplementary Materials

The following are available online at https://www.mdpi.com/article/10.3390/ma14133683/s1, Supplemental Figure S1: Integrated powder X-ray diffraction patternsof cerium dioxideon compression pastthe Fm-3m to Pnam transition nominally at 31 GPa. Only a little conversion occurs up to 37.7 GPa with someof thelow-pressurephase persistingup to the highest pressures.

## Abbreviation

PTM	Pressure Transmitting Medium
