# Supplementary material for: High Pressure Brillouin Spectroscopy and X-ray Diffraction of Cerium Dioxide
Source: Materials (Basel). 2021 Jul 1;14(13):3683. doi: 10.3390/ma14133683 (PMC8269805; doi:10.3390/ma14133683)
Supplement: Supplementary file 1 [file materials-14-03683-s001.zip › materials-1266601-supplementary.pdf]

Supplemental Materials – High Pressure Brillouin Spectroscopy and X-ray Diffraction of Cerium Dioxide

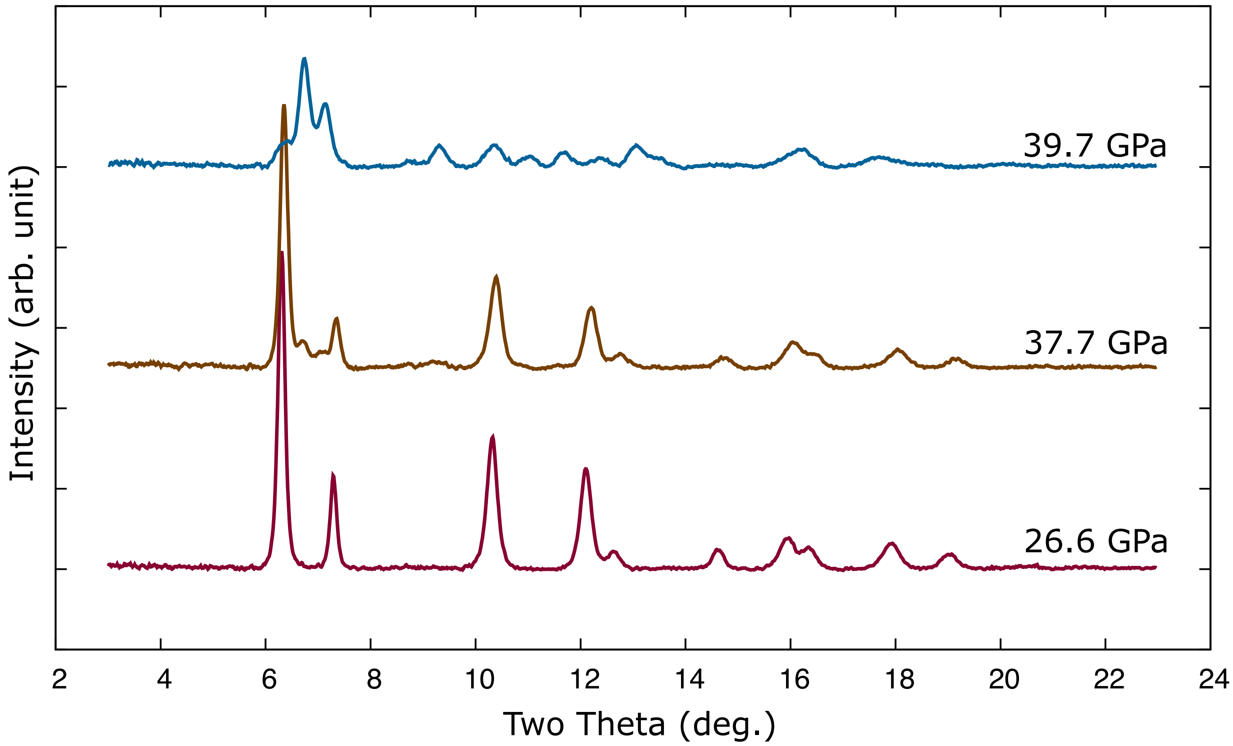

Supplemental Figure 1: Integrated powder x-ray diffraction patterns of cerium dioxide on compression past the Fm-3m to Pnam transition nominally at 31 GPa. Only a little conversion occurs up to 37.7 GPa with some of the low-pressure phase persisting up to the highest pressures.
